# Spatial Access to Emergency Services in Low- and Middle-Income Countries: A GIS-Based Analysis

**DOI:** 10.1371/journal.pone.0141113

**Published:** 2015-11-03

**Authors:** Gavin Tansley, Nadine Schuurman, Ofer Amram, Natalie Yanchar

**Affiliations:** 1 Department of Community Health and Epidemiology, Dalhousie University, Halifax, Nova Scotia, Canada; 2 Department of Geography, Simon Fraser University, Burnaby, British Columbia, Canada; Kenya Medical Research Institute - Wellcome Trust Research Programme, KENYA

## Abstract

Injury is a leading cause of the global disease burden, accounting for 10 percent of all deaths worldwide. Despite 90 percent of these deaths occurring in low and middle-income countries (LMICs), the majority of trauma research and infrastructure development has taken place in high-income settings. Furthermore, although accessible services are of central importance to a mature trauma system, there remains a paucity of literature describing the spatial accessibility of emergency services in LMICs. Using data from the Service Provision Assessment component of the Demographic and Health Surveys of Namibia and Haiti we defined the capabilities of healthcare facilities in each country in terms of their preparedness to provide emergency services. A Geographic Information System-based network analysis method was used to define 5- 10- and 50-kilometer catchment areas for all facilities capable of providing 24-hour care, higher-level resuscitative services or tertiary care. The proportion of a country’s population with access to each level of service was obtained by amalgamating the catchment areas with a population layer. A significant proportion of the population of both countries had poor spatial access to lower level services with 25% of the population of Haiti and 51% of the population of Namibia living further than 50 kilometers from a facility capable of providing 24-hour care. Spatial access to tertiary care was considerably lower with 51% of Haitians and 72% of Namibians having no access to these higher-level services within 50 kilometers. These results demonstrate a significant disparity in potential spatial access to emergency services in two LMICs compared to analogous estimates from high-income settings, and suggest that strengthening the capabilities of existing facilities may improve the equity of emergency services in these countries. Routine collection of georeferenced patient and facility data in LMICs will be important to understanding how spatial access to services influences outcomes.

## Introduction

Injury is recognized as being a major contributor to the global disease burden, accounting for approximately 1 out of every 10 deaths in the most recent World Health Organization (WHO) estimates [1]. Although decades of research have led to the development of effective emergency services designed to reduce the morbidity and mortality associated with trauma, the progress has been largely limited to developed countries where only 10% of injury-related deaths occur [2–5]. This disparity in emergency services access, availability and quality prompted the World Health Assembly (WHA) to pass Resolution 60.22 in 2007 [6]. This document supported the effectiveness of improving trauma and emergency care services, and urged WHO member states to develop 10 specific areas deemed essential to improving these services [6,7]. Identified in this resolution was the need to “assess comprehensively the prehospital and emergency care context including, where necessary, identifying unmet need”[6]. Despite this resolution, there remains a paucity of studies assessing emergency care services in low and middle-income countries (LMICs). Several studies have evaluated aspects of trauma care services in a subset of mostly African countries, but these studies have focused almost exclusively on infrastructure and personnel, leaving issues concerning spatial access to services largely unstudied [8,9].

For decades timely access to trauma services has been recognized as an important pillar of a mature trauma system, supported by several studies demonstrating a survival disadvantage when definitive care is delayed [10,11]. This observation has been popularized as “the golden hour” of trauma, implying that delays to definitive care of greater than one hour are associated with worse outcomes. Although the heterogeneity of traumatic injury impedes the generalizability of the golden hour, the benefits of prompt access to definitive care are robust, and “the golden hour” remains a reasonable benchmark for developing trauma systems [12]. Defining populations at greater distances from tertiary care is an integral step towards improving trauma care, either through infrastructure development, inter-facility transfer agreements or the utilization of telemedicine techniques. To date, assessments of spatial access to trauma care have been carried out in both Canada and the United States with comparable results, but there is currently a lack of studies examining spatial access to trauma services in LMICs [13,14]. By identifying inequalities in access to emergency services in LMICs, it may be possible to more intelligently focus infrastructure development in these nations.

Here we describe the use of a Geographic Information System (GIS) network analysis method to quantify the population-level spatial accessibility to facilities capable of providing emergency trauma services for the populations of two LMICs, Namibia and Haiti.

## Setting

### Namibia

Namibia is a sparsely populated country situated in southwestern Africa, with an estimated population of approximately 2.3 million people concentrated at a density of 2.7 persons per km^2^ [15]. Although the population of Namibia is focused in the North of the country, two-thirds of the population lives in rural areas, generating unique challenges for health services delivery [16]. According to the 2011 census, the average life expectancy is 63 years, with an infant mortality rate of 33.5 deaths per 1,000 live births [15]. Classified as an upper middle-income country by the World Bank, Namibia experiences significant disparities in wealth distribution, which contribute to the health indicators more reminiscent of lower income nations. The health system of Namibia is divided into 13 administrative areas and 34 health districts. The district hospitals provide the majority of inpatient services and are supported by three intermediate hospitals and one national referral centre [16]. In addition to these public services, public-private partnerships, faith-based organizations and non-governmental organizations (NGOs) all contribute to service provision [16].

### Republic of Haiti

Located in the Caribbean, Haiti has a population of 10.2 million people living at a density of 366.6 persons/km^2^ [15]. In addition to being a low-income country, Haiti continues to experience the social and economic consequences of a 2010 earthquake. With an average life expectancy of 63 years, and an infant mortality rate of 40.2 per 1,000 live births the health indicators of Haiti are similar to many other developing nations [15]. Haiti is divided into ten administrative departments with a health system comprised of primary secondary and tertiary levels. Each of these levels serves a successively larger geographic area with generally increasing comprehensiveness. The public sector is further supported by private, faith-based, and NGO-run centres, but the capabilities of the individual facilities vary significantly [17].

## Methods

### Health Facilities

Data on health facility capabilities and locations were obtained from the Service Provision Assessments (SPAs) of Haiti and Namibia, generated by the Demographic and Health Surveys Program of the United States Agency for International Development [16–18]. The SPA is a health facility assessment that provides a comprehensive audit of the infrastructure, personnel, and service provision readiness of a nation’s health facilities. On-site interviewing teams, typically composed of 3–4 trained health workers, conducted these audits by administering a standardized inventory questionnaire to the most knowledgeable person available at the time of the visit. Additionally, interviewers verified the existence of assessed items by confirming their presence in the facility during the interview. The SPAs of Haiti and Namibia both included non-displaced geocodes for facility location obtained by the interviewing teams using Global Positioning Systems. Furthermore, these SPAs attempted to survey all operational facilities in their respective countries. In Namibia, of the 446 facilities identified from a master facility list (46 hospitals, 49 health centres, 327 clinics, 15 voluntary testing and counseling centres and 9 sick bays), 92% were successfully audited during the most recent SPA in 2009, 2% were found to be duplicates, 3% were closed and 2% were found to be non-existent or not suitable for assessment. In Haiti, of the 1080 facilities identified 84% were successfully audited during the most recent SPA in 2013 (121 hospitals, 129 health centres with beds, 298 health centres without beds, and 359 dispensaries), 8.1% were not found, and 8.0% were closed. From the SPA data, facilities were classified into Levels A, B, or C depending on their capacity to provide 24-hour care, resuscitative services, or definitive trauma services, respectively. Facilities were assigned to these categories using pre-defined criteria based on available infrastructure, equipment and personnel (Table 1). The criteria used were derived from the WHO’s Integrated Management for Emergency and Essential Surgical Care Toolkit [19]. Facilities that did not have the capacity to provide any of these services were classified as “Level X” and excluded from further analysis.

**Table 1:** Facility classification criteria adapted from the WHO Integrated Management of Emergency and Essential Surgical Care Toolkit [19].

Facility Level	Description	Criteria
**Level A**	Facilities with 24-hour emergency services	Overnight beds, 24-hour duty schedule, at least 2 qualified providers*, availability of water and electricity, a functioning telephone, on-site latrines
**Level B**	Facilities with resuscitative capabilities	Satisfies all Level A criteria, availability of IV fluids, blood products, basic surgical instruments**, local anesthesia, sterile dressings, supplemental oxygen
**Level C**	Tertiary care facilities	Tertiary hospital designation or any facilitiy that satisfies all Level B criteria, has >50 inpatient beds and a surgeon on staff.
**Level X**	Facilities with insufficient capacity to provide emergency services	Does not satisfy the criteria to qualify for any of Levels A, B, or C

* qualified providers were defined as specialist physicians, medical officers, or nurses.

** basic surgical instruments included forceps, a needle driver, and sterile scissors.

### Population Data

National population data for Haiti and Namibia were represented by a 100 square meter gridded population surface generated by the WorldPop project [20]. This open access initiative combines census data with ancillary datasets such as settlement locations and land use to estimate the number of persons residing within each 100 square meter grid cell. As the majority of a census enumeration area’s population resides within settlements, this method provides higher resolution approximations of the spatial distribution of populations than estimates based exclusively on the census-derived population counts of administrative areas [21]. For Haiti, the dataset was built using the most recent census in 2009, with modeled estimates for 2015 based on United Nations (UN) population projections. Similarly, the Namibia dataset was built using the 2011 census, and updated based on 2015 UN projections. The populations of regions and enumeration areas were obtained by aggregating the population surface with first and fourth level administrative boundary layers obtained from the governments of the study nations or an online data repository [22,23].

### Road Networks

Publically available national road networks of Namibia and Haiti were obtained through the Google Map Maker project (Google, Mountain View, CA); a crowd sourced mapping initiative that generates digitized maps through the use of aerial imagery [24].

### Network Analysis

The national road networks of Haiti and Namibia were imported into ArcMap 10.2 (Esri, Redlands, CA) for network dataset construction prior to performing network analyses. Network analysis is a vector-based routing technique that uses road network data, represented by a series of line segments and connecting nodes, to calculate travel costs between two points [13]. The topologies of the road networks were corrected to ensure all intersections were composed of only endpoint nodes, and road segment lengths were subsequently calculated following projection into the appropriate Universal Transverse Mercator coordinate system (33S, and 18N for Namibia and Haiti, respectively).

The resulting network datasets were individually loaded into ArcMap in addition to layers containing the point locations of each facility. The 5-, 10-, and 50-kilometer service areas for each facility were generated using ArcMap’s Network Analyst extension. This resulted in three overlapping service areas for each location composed of all road segments within 5-, 10-, and 50-kilometers of the originating facility. Each catchment area was exported to a new layer designated by the corresponding facility level and the service area size. Populations were attributed to a facility by creating a 1000-meter buffer around each facility’s service area and calculating the sum of all population cells within the buffer. This buffer size was chosen to capture all individuals residing within a reasonable distance of serviced roads acknowledging the probable presence of small tracks or trails not present in the digitized road network. The proportion of each administrative region’s population with access to Level A, B, and C facilities was calculated by extracting only the population cells within each buffered service area and calculating the sum of the cells spatially located within each administrative region. This value was then expressed as a proportion of the region’s total population.

### Sensitivity Analysis

To evaluate the stability of the estimates over a range of buffer sizes, the size of the 1000-meter buffer was augmented by ± 50% to create two additional service areas for each facility. The population calculations were repeated with the augmented service areas to generate a lower and upper limit for the population estimates, designated as an uncertainty interval (UI).

## Results

### Namibia

**Fig 1:**
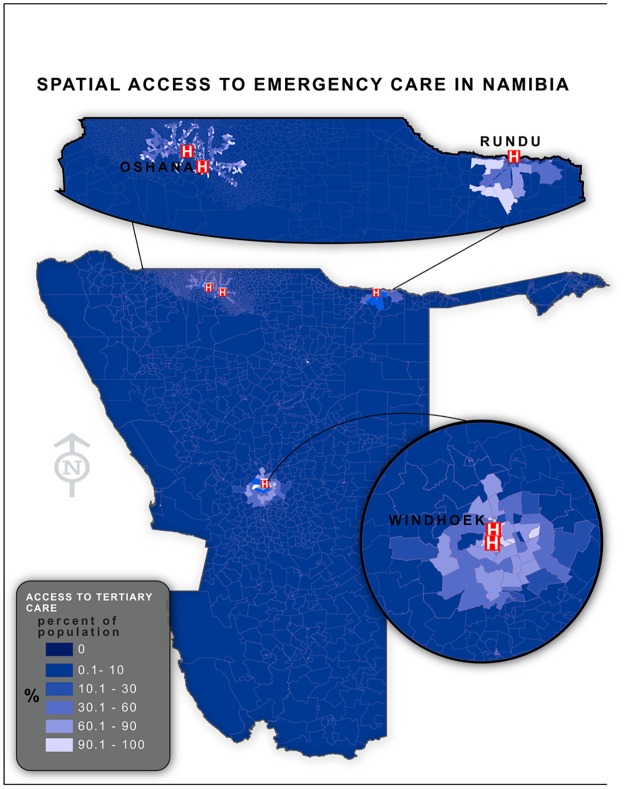
Population-level spatial access to tertiary care in Namibia. Results from network analysis demonstrating the proportion of each census enumeration area’s population with spatial access to tertiary care within 50 kilometers of their residence.

Of the 410 facilities found to be operational in Namibia at the time of the SPA, 12.4%, 7.3%, and 1.2% were found to be capable of providing level A, B, and C care, respectively. 88% of facilities were found to be unsuitable for providing emergency care and were designated as level X. From these subsets of facilities, it was found through our network analysis that 28% (UI 24.2–29.8%) of the population was within 50km of road travel distance to tertiary care (Table 2 and Fig 1). The results for the additional facility levels and service area sizes are described in Table 2.

**Table 2:** Population-level spatial access to Level A, B, and C facilities in Haiti and Namibia.

	Level A			Level B			Level C		
	5 Km	10 Km	50 Km	5 Km	10 Km	50 Km	5 Km	10 Km	50 Km
Haiti	53.1	61.9	75.1	24.6	32	49.4	29.6	34.6	48.8
	(48.3–56.6)	(55.1–66.5)	(64.6–81.0)	(23.5–25.7)	(30.6–32.7)	(44.4–52.3)	(27.6–30.9)	(33.5–35.3)	(44.6–51.1)
Namibia	29.1	34.7	48.7	26.5	32.5	43.2	16.3	21.7	27.7
	(24.5–31.8)	(30.9–37.0)	(41.5–53.0)	(22.2–29.0)	(29.2–34.4)	(37.5–46.9)	(13.3–18.1)	(19.7–22.6)	(24.2–29.8)

Proportion of the Haitian and Namibian populations within 5-, 10-, and 50-kilometer service areas of Level A, B, and C facilities. Numbers in parentheses represent the uncertainty intervals obtained by augmenting the size of the service area buffer by ± 50%.

By identifying the proportion of a geographic region’s population captured within a facility’s service area, it was possible to determine if there was differential access to services by region. Unsurprisingly, there was substantial variability in regional spatial access to emergency services with the proportion of a region’s population within a 50-km service area of a Level C facility ranging from 0% in the more remote regions to 95.4% in the most urban region (Table 3).

**Table 3:** Spatial access to level A, B, and C facilities in Namibia, by region.

Region	Level A			Level B			Level C		
	5 Km	10 Km	50 Km	5 Km	10 Km	50 Km	5 Km	10 Km	50 Km
Caprivi	0	0	0	0	0	0	0	0	0
	(N/A)	(N/A)	(N/A)	(N/A)	(N/A)	(N/A)	(N/A)	(N/A)	(N/A)
Erongo	70.1	71.6	79	69.9	71.6	79.0	0	0	0
	(67.1–71.1)	(69.2–72.6)	(75.3–80.4)	(66.5–70.9)	(69.1–72.6)	(75.3–80.4)	(N/A)	(N/A)	(N/A)
Hardap	12.9	14.5	20	11.4	12.9	16.7	0	0	0
	(9.1–14.5)	(10.5–15.9)	(15.4–21.8)	(7.9–13.0)	(9.3–14.2)	(12.6–18.3)	(N/A)	(N/A)	(N/A)
Karas	34.7	36.3	40.9	33.7	35.1	38.1	0	0	0
	(32.7–35.4)	(34.4–36.9)	(38.3–41.8)	(31.8–34.3)	(33.4–35.7)	(35.9–38.8)	(N/A)	(N/A)	(N/A)
Kavango	5.3	9.6	56.9	2.1	4.2	22.3	14.7	21.5	33.3
	(3.8–6.6)	(7.1–11.6)	(41.9–64.0)	(1.5–2.6)	(3.1–4.9)	(17.8–25.0)	(8.4–18.4)	(13.8–24.3)	23.2–37.7)
Khomas	81.4	93.8	95.5	74.9	93.4	95.4	74.5	93.4	95.4
	(71.5–86.0)	(92.8–94.2)	(94.4–95.9)	(64.5–80.5)	(92.2–93.8)	(94.3–95.8)	(64.4–80.6)	(92.2–93.8)	(94.3–95.8)
Kunene	4.8	5.2	8.7	4.7	5.2	8.8	0	0	0
	(2.9–5.4)	(3.1–5.7)	(6.3–9.5)	(2.8–5.4)	(3.2–5.7)	(6.3–9.5)	(N/A)	(N/A)	(N/A)
Ohangwena	5.8	10.3	31.2	4.0	8.6	29.9	0	0	13
	(3.6–7.7)	(7.0–12.8)	(21.5–38.5)	(2.6–5.4)	(5.8–10.6)	(20.2–37.6)	(N/A)	(N/A)	(8.4–17.3)
Omaheke	16.5	19	28.1	13.8	16.0	21.7	0	0	0
	(11.9–20.4)	(15.0–22.8)	(22.4–32.4)	(9.8–17.2)	(12.2–19.5)	(17.0–25.4)	(N/A)	(N/A)	(N/A)
Omusati	10.9	15.2	33.3	7.4	9.8	27.2	0	0	7.5
	(8.4–12.3)	(11.7–17.5)	(25.1–39.3)	(5.7–8.2)	(7.5–11.0)	(20.4–32.4)	(N/A)	(N/A)	(5.3–9.1)
Oshana	26.3	38.1	53.6	25.0	37.9	53.6	21.2	37.1	53.7
	(19.1–30.7)	(27.7–43.9)	(39.5–62.4)	(18.0–29.3)	(27.6–43.8)	(39.5–62.4)	(15.2–25.0)	(27.0–42.6)	(39.8–62.4)
Oshikoto	15.5	20.7	29.8	12.3	15.0	24.6	4.2	6.4	14.3
	(11.3–18.6)	(15.5–24.5)	(22.6–34.6)	(9.1–14.5)	(11.2–17.3)	(18.6–28.5)	(2.9–5.4)	(4.6–7.7)	(10.6–17.2)
Otjozondjupa	26.1	29.3	42.1	25.8	28.8	40.6	0	0	0.1
	(17.5–32.4)	(20.3–35.5)	(31.4–49.2)	(17.4–32.5)	(20.0–35.1)	(29.9–47.5)	(N/A)	(N/A)	(0.1–0.1)

Proportion of each region’s population within 5-, 10-, and 50-kilometer service areas of Level A, B, and C facilities. Numbers in parentheses represent the uncertainty intervals obtained by augmenting the size of the service area buffer by ± 50%.

### Haiti

**Fig 2:**
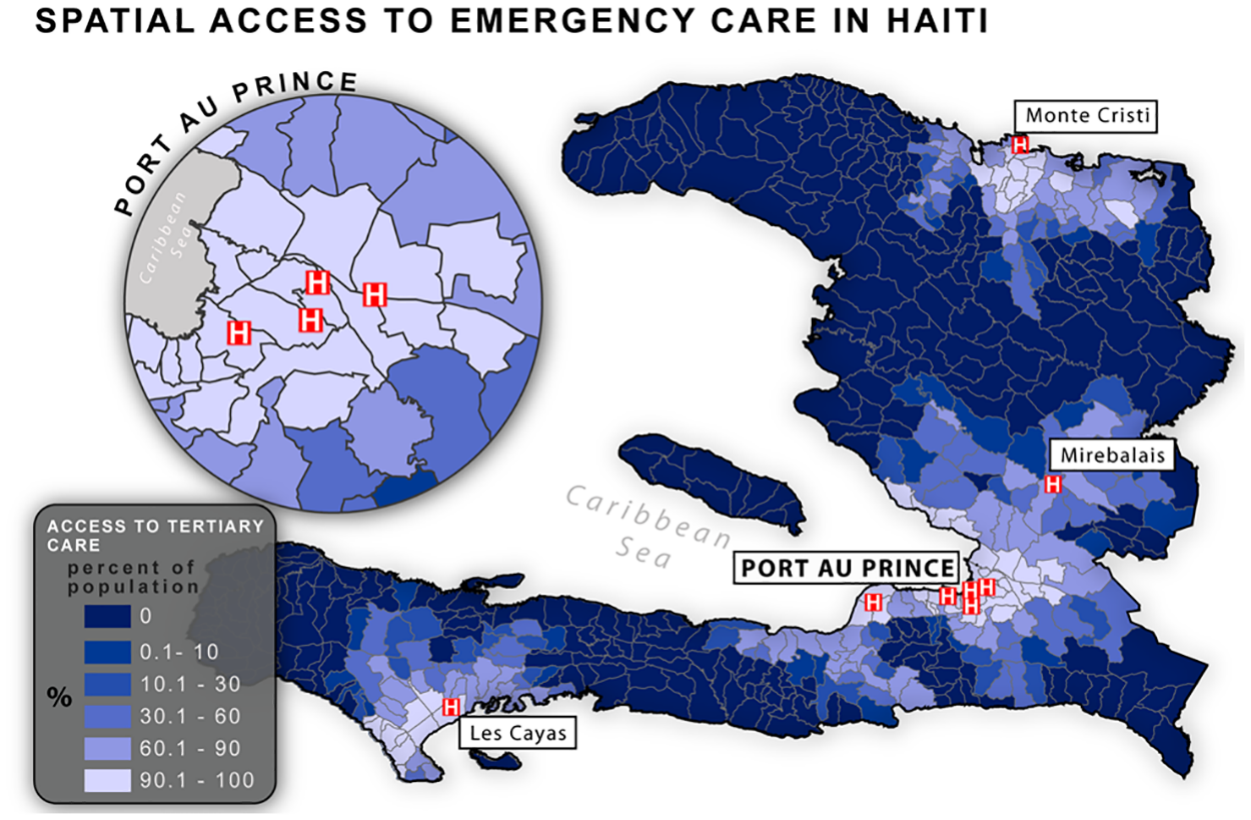
Population-level spatial access to tertiary care in Haiti. Results from network analysis demonstrating the proportion of each census enumeration area’s population with spatial access to tertiary trauma care within 50 kilometers of their residence.

In Haiti, 907 facilities were found to be operational at the time of the SPA assessments. Of these, 18.9%, 1.7%, and 0.9% of facilities were found to have the capacity to provide Level A, B and C care, respectively. 81.1% of facilities were designated as Level X. Overall, 48.8% (UI 44.6–51.1%) of the population was found to live within a 50 km catchment of a Level C facility (Fig 2, Table 2). Access to services was similarly variable across geographic regions, with Level C access ranging from 0% in the Nord Ouest department to 89.1% in the Ouest department, the nation’s most populous area (Table 4).

**Table 4:** Spatial access to level A, B, and C facilities in Haiti, by department.

Department	Level A			Level B			Level C		
	5 Km	10 Km	50 Km	5 Km	10 Km	50 Km	5 Km	10 Km	50 Km
Ouest	83.6	88.7	92.3	58.3	73.1	87.5	64.8	75.2	89.1
	(81.1–85.5)	(85.4–90.2)	(88.1–94.6)	(56.0–60.6)	(71.3–74.2)	(84.3–88.8)	(60.9–67.4)	(73.6–76.2)	(85.6–90.4)
Sud-Est	22.3	31.7	57.2	0	0	7.2	0	0	21.4
	(18.3–25.6)	(24.1–37.2)	(41.8–67.2)	(N/A)	(N/A)	(4.3–9.3)	(N/A)	(N/A)	(17.2–24.6)
Nord	52.9	65.5	73.7	0	0	8.7	28.5	32	60.7
	(44.6–57.9)	(54.8–71.3)	(61.1–79.9)	(N/A)	(N/A)	(6.2–9.8)	(25.8–29.6)	(30.6–32.9)	(51.9–65.3)
Nord-Est	38.2	55.1	71.4	0	0	1.9	0	0	23.9
	(30.9–44.0)	(43.0–62.3)	(53.8–80.1)	(N/A)	(N/A)	(1.3–2.4)	(N/A)	(N/A)	(17.3–27.5)
Artibonite	34.7	47.4	72.0	1.1	6.3	37.3	0	0	2.0
	(28.5–39.0)	(38.5–53.3)	(59.1–79.6)	(0.4–1.6)	(4.9–7.2)	(29.5–41.8)	(N/A)	(N/A)	(1.2–2.9)
Centre	15.4	24.3	45.2	5.9	9.3	38.2	3.0	5.0	20.3
	(10.5–19.4)	(15.8–30.7)	(28.7–56.4)	(4.8–6.8)	(6.8–11.1)	(24.6–47.5)	(2.0–3.7)	(3.1–6.6)	(12.7–25.5)
Sud	29.8	45.3	63.4	10.2	16	59.4	10.0	15.5	50.6
	(21.8–36.1)	(32.5–53.9)	(47.2–72.2)	(9.1–11.1)	(13.1–18.1)	(44.6–67.6)	(7.8–11.1)	(12.7–17.5)	(38.8–57.1)
Grande-Anse	18.3	28.0	52.3	0	0	1.8	0	0	2.8
	(13.5–22.4)	(19.8–34.1)	(35.7–62.8)	(N/A)	(N/A)	(1.0–2.5)	(N/A)	(N/A)	(1.5–3.6)
Nippes	23.9	36.3	51.2	0	0.1	27.6	0	0	2.1
	(15.1–31.1)	(23.1–45.9)	(33.0–63.0)	(N/A)	(0.0–0.3)	(18.2–34.2)	(N/A)	(N/A)	(1.2–3.0)
Nord-Ouest	34.9	39.2	55.6	0	0	0	0	0	0
	(29.4–39.1)	(32.6–43.7)	(43.9–62.5)	(N/A)	(N/A)	(N/A)	(N/A)	(N/A)	(N/A)

Proportion of each department’s population within 5-, 10-, and 50-kilometer service areas of Level A, B, and C facilities. Numbers in parentheses represent the uncertainty intervals obtained by augmenting the size of the service area buffer by ± 50%.

## Discussion

Studies examining spatial access to trauma services for North American populations have identified that 78% of Canadians and 84% of Americans have access to tertiary trauma care within 1-hour of driving time [13,14]. This study provides the first national level, GIS-based analysis describing spatial access to emergency services in two LMICs. Because of a lack of data available on road surfaces and speed limits, a distance-based service area was used in this study. Although this limits our ability to make direct comparisons to prior studies, a 50-km service area was chosen as an estimate of the maximum travelable distance within a one hour prehospital interval, based on the high proportion of unpaved roads in both countries (86% in Namibia, 82% in Haiti), and the underdeveloped prehospital systems in most LMICs [25,26]. Despite these methodological differences, 72.3% of the Namibian population and 51.2% of the Haitian population live further than 50 km from Level C facilities capable of providing tertiary care, suggesting a high proportion of both populations may be at elevated risk of poorer outcomes following major trauma. As the majority of capable facilities are located in more populated urban areas, rural populations appear particularly disadvantaged in both Haiti and Namibia.

Prolonged prehospital times following major trauma have previously been associated with worse outcomes in some high-income settings. A study by Sampalis et al conducted in the Canadian province of Quebec demonstrated a 3-fold increased odds of dying in patients with prehospital times longer than 1 hour [10]. This finding was later replicated by the same group when they demonstrated that a reduced prehospital time was associated with a reduced odds of dying after controlling for injury severity and age [11]. Although two large American studies failed to replicate any association between prehospital time and outcome, a recent meta-analysis suggests there is a benefit of short prehospital intervals for individuals with head injuries and hemodynamically unstable victims of penetrating trauma [26–28]. Although none of this work took place in LMICs, the disproportionately high number of prehospital deaths observed in low-income settings suggests spatial analysis-driven improvements to the organization of post-injury care may be an effective way to reduce prehospital times and improve survival following major trauma [29,30].

Strategies such as helicopter transport [31] have been utilized in high-income settings to improve access to services for populations injured in rural areas, but the feasibility of this strategy in LMICs would be substantially limited by financial constraints. The 81% of facilities in Haiti, and the 88% of facilities in Namibia found to be incapable of providing even basic emergency services suggests that improving the supplies, personnel and infrastructure gap may be a more cost effective means of improving access to these services. A study by Hsia et al found a similar number of facilities lacking the capacity to provide emergency services when they examined the personnel and infrastructure of the healthcare systems in five Sub-Saharan African countries [8]. Although many of the facilities in this study were classified as dispensaries and shouldn’t be expected to provide emergency services, many others simply lacked the basic and inexpensive equipment necessary to provide emergency care. Addressing this supplies and infrastructure gap by systematically identifying and upgrading the facilities expected to provide the greatest improvements in population-level spatial access to services is a feasible approach to improving trauma care in LMICs, and has recently been identified as a key objective in the field of global surgery [32].

GIS-based studies are being conducted with increasing frequency in LMICs due, in part, to the increasing ubiquity of high quality spatial data. The availability of digitized road networks has facilitated many of these studies by allowing spatial access to be quantified based on established transport networks as opposed to straight-line distances, which have been shown to be less accurate proxies for spatial access in LMICs [33,34]. Two recent studies utilized raster-based cost surfaces built using road networks to model access to health services in LMICs [35,36]. These methods have been previously demonstrated to provide comparable results to the network analysis approach taken in this study [37].

The road networks used in this analysis were of sufficient quality to allow the utilization of network analysis methods, but the presence of roads or paths that remain unmapped could potentially result in individuals being erroneously excluded from a service area. However, when we conducted the same analysis using service areas created with 5-, 10- and 50 km Euclidean buffers around facilities, similar results were obtained (S1 Table). This finding suggests the factors responsible for the poor spatial access to trauma care in these countries is more likely related to the facilities’ capabilities as opposed to the national road networks servicing the various catchments.

This study used population distributions and facility locations to estimate potential spatial access to trauma care in Namibia and Haiti. As there are currently no data available on facility utilization at the national level in either of these countries, the estimates provided are not empirically validated. It is possible that a facility deemed capable of providing trauma care may not be utilized by a patient for non-spatial reasons including cost or personal preferences, thereby influencing revealed access to services. However, with acute conditions such as injury, these issues would likely be minimized by the recognized need for prompt care, resulting in the utilization of the nearest facility. Additionally, as 25% of global injuries result from motor vehicle incidents, the use of residences as a proxy for the locations of demand for trauma care may be invalid, further highlighting the need for georeferenced injury location data in LMICs [1].

The use of SPA assessments to define a facility’s capabilities is limited by the fact that they offer only a snapshot of each facility. Changes in resources and infrastructure over time have the potential to alter the healthcare landscape of these countries, resulting in changes in spatial access to care. Because we utilized very basic personnel and infrastructure criteria, our estimates of facilities’ capabilities are likely conservative, and are expected to remain similar despite day-to-day variability in supplies and equipment availability. In several instances, a facility that was defined as a tertiary facility by the government was not included in the analysis of the lower level facilities because the facility had no access to basic infrastructure or supplies such as water or blood products, and therefore failed to meet inclusion criteria. Although this occurred in only three instances in our analysis, it underscores the contrast between tertiary care in high- and low-income settings as well as the difficulties associated with standardizing analyses in LMICs.

This study highlights significant inequalities in spatial access to trauma services in two LMICs. Although Haiti and Namibia are distinct in terms of geography, population and healthcare infrastructure, caution must be exercised before extrapolating these results to other low-income settings. This study demonstrates the importance and utility of geocoded data in LMICs, and suggests GIS-based methods could be useful in trauma system development to help direct infrastructure expansion. Subsequent research should be focused on obtaining geocoded utilization data for victims of trauma in LMICs to verify studies of potential spatial access.

## Supporting Information

S1 Table Percentage of the Namibian and Haitian populations within a 50-km radius of Level A, B, and C care. Proportion of the population of Namibia and Haiti residing within a 50-kilometer Euclidean buffer around each facility
(XLSX)
